# Is There Any Cardiovascular Concern Regarding the Use of Aromatase Inhibitors in Breast Cancer?

**DOI:** 10.5935/abc.20190092

**Published:** 2019-05

**Authors:** Tatiana F. G. Galvão

**Affiliations:** Hospital Israelita Albert Einstein, São Paulo, SP - Brazil

**Keywords:** Aromatase Inhibitors, Breast Neoplasms, Cholesterol, Blood Glicose, Estrogen Replacement, cardiovascular Diseases/prevention & control

Breast cancer is the most frequent malignancy in women worldwide, and the second leading cause of cancer mortality.^1^ Due to advances in prevention, early detection and treatment, breast cancer mortality has decreased by nearly 40% during the last four decades.^1^ However, this optimistic scenario has been counterbalanced by an increasing risk from cardiovascular disease (CVD) in breast cancer survivors. Indeed, CVD is a leading cause of mortality in breast cancer survivors.^2-5^ The mechanisms by which these patients are at increased cardiovascular risk are multiple ones, including the effects of cancer itself (inflammation, oxidative stress, prothrombotic status, autonomic dysfunction, etc.) or due to the side effects related to chemotherapy and radiotherapy (metabolic dysfunction, cardiotoxicity).^6^ Some of these pathways may have prognostic significance: a previous investigation showed that autonomic modulation in breast cancer patients is an independent predictor of cardiovascular risk.^6^

In this issue of the *Arquivos Brasileiros de Cardiologia*, the study "Changes in Cardiac Autonomic Modulation in Women with Breast Cancer Using Aromatase Inhibitors and the Relation with Biochemical Variables"^7^ was aimed to explore not breast cancer but the associations of one of its treatments (namely aromatase inhibitors, AI) with markers of autonomic dysfunction, as well as metabolic and inflammatory parameters in postmenopausal women. The rationale for exploring this specific therapy was based on a recent meta-analysis showing that prolonged AI use had a marginally effect of having a CVD event (odds ratio: 1.18, 95% CI = 1.00-1.40) as compared to placebo.^8^

In the current investigation,^7^ the authors performed a cross-sectional analysis comparing two groups of participants: 1) women with breast cancer, treated with AIs and 2) postmenopausal women without breast cancer. For the evaluation of the autonomic modulation, heart rate was recorded beat-to-beat for 30 minutes and the series of RR intervals obtained were used to calculate rate variability indices (RVI): mean RR ms, SDNN (standard deviation of all normal RR intervals, expressed in milliseconds) ms, mean Heart Rate (HR), RMSSD (square root of the mean of the squared differences between adjacent normal RR interval) ms, NN50 (number of pairs of successive NNs that differ by more than 50 ms) count, p NN 50% (proportion of NN 50 divided by total number of NNs), RRtri (RR triangular), TINN (triangular interpolation of NN interval ) ms, SD1ms, SD2 ms, LF (low frequency) ms^2^, HF(high frequency) ms^2^. LF; HF ms^2^. Despite some criticism, all these parameters provide an indirect evaluation of autonomic function. In addition, the following metabolic and inflammatory parameters were analyzed: fasting glycemia, triglycerides. HDL-cholesterol and C-reactive protein (CRP). The study showed that lower values of HR variability indices were observed in breast cancer patients in relation to the control group. Besides, there was an inverse correlation between the indices SDNN, SD2 and HFms with triglycerides. No statistically significant correlations were found between HR variability indices and other biochemical variables.

While this study is timely, addressing the increasing awareness of the cardiovascular effects attributed to cancer and its treatments, there are significant limitations that deserve an appropriate discussion. This is a small cross-sectional study addressing autonomic and cardiovascular parameters in patients already on AI treatment. The lack of baseline measurements (before starting AI therapy) prevent any conclusion from being drawn on whether the main results were related to AIs, the breast cancer per se or some other residual factor. Regarding the latter, the authors did not evaluate the potential role of important confounding factors (such as hypertension or chronic use of medications) that could affect autonomic modulation or the cardiovascular biomarkers.

Having said that, this study raised more questions than answers, but certainly stimulates additional investigations testing the cardiovascular safety of this important chemotherapy class. In the past decade, AIs have been the recommended first-line adjuvant endocrine therapy in postmenopausal women with hormone receptor-positive breast cancer; they are associated with improved disease-free survival and overall survival.^9^ Based on the increasing prevalence of breast cancer worldwide and the related burden of survivors, it is crucial to clarify the cardiovascular safety of the related drugs. The benefits of cancer treatment should be balanced with the presence and magnitude of severe side effects - including cardiovascular events - in the long-term follow-up.
